# Theoretical Analysis on Heteroleptic Cu(I)-Based Complexes for Dye-Sensitized Solar Cells: Effect of Anchors on Electronic Structure, Spectrum, Excitation, and Intramolecular and Interfacial Electron Transfer

**DOI:** 10.3390/molecules25163681

**Published:** 2020-08-12

**Authors:** Zhijie Xu, Xiaoqing Lu, Yuanyuan Li, Shuxian Wei

**Affiliations:** 1 College of Science, China University of Petroleum, Qingdao 266580, China; yylzjx@163.com; 2 School of Materials Science and Engineering, China University of Petroleum, Qingdao 266580, China

**Keywords:** dye-sensitized solar cells (DSSCs), Cu(I)-based complex, anchor, density functional theory (DFT)

## Abstract

Two groups of heteroleptic Cu(I)-based dyes were designed and theoretically investigated by density functional theory (DFT) and time-dependent DFT (TD-DFT) methods. Different anchors were integrated into the dye skeleton to shed light on how the type of anchor influenced the electronic structure, absorption spectrum, electron excitation, and intramolecular and interfacial electron transfer of dyes. The results indicated that, compared with other dyes, the dyes with cyanoacrylic acid and nitric acid exhibited more appropriate electron distributions in frontier molecular orbitals (FMOs), lower HOMO (the highest occupied molecular orbital) –LUMO (the lowest unoccupied molecular orbital) energy gaps, broader absorption spectral ranges as well as improved spectral characteristics in the near-infrared region and better intramolecular electron transfer (IET) characteristics with more electrons transferred to longer distances, but smaller orbital overlap. Among all the studied Cu(I)-based dyes, B1 and P1 (with cyanoacrylic acid anchoring group) exhibited the best interface electronic structure parameters with a relatively short electron injection time (τ_inj_) and large dipole moment (μ_normal_), which would have a positive effect on the open-circuit photovoltage (V_oc_) and short-circuit current density (J_sc_), resulting in high power conversion efficiency (PCE) of dye-sensitized solar cells (DSSCs). Our findings are expected to provide a new insight into the designing and screening of high-performance dyes for DSSCs.

## 1. Introduction

Dye-sensitized solar cells (DSSCs), as a promising photovoltaic device, with advantages of relatively high efficiency and low-cost fabrication, have gained widespread attention since Ru(II) polypyridyl complexes were first reported and their excellent power conversion efficiency (PCE) was demonstrated in 1991 by Grätzel and co-workers [1]. Since then, lots of significant progress has been made in Ru(II) polypyridyl complex-based DSSCs, with PCE up to 11.5% [2,3,4,5,6,7,8,9]; however, the rarity and high cost of ruthenium limited the large-scale utilization of Ru(II)-based dyes in DSSC application. Therefore, it is necessary to develop novel dyes with plentiful and cheap components as other replacements. Copper, as an abundant and nontoxic, non-noble metal, might be a good substitute for ruthenium in DSSCs. Sauvage and co-workers first introduced heteroleptic Cu(I)-polypyridine complexes in DSSCs in 1994 [10]. Furthermore, it was reported that Cu(I)-based complexes could be efficient dyes due to their adequate electron transfer capacities in DSSCs [11,12]. In recent years, many molecular engineering strategies were applied in Cu(I)-based complexes for DSSC application and impressive photoconversion efficiencies were obtained. In 2008, Constable and co-workers reported that 6,6′-disubstituted-2,2‘-bipyridine-based Cu(I) complexes with carboxylic acid as the anchoring group can be used as effective sensitizers for TiO_2_ and surprisingly resulted in high PCE of 2.3% [13]. A few years later, PCE of Cu(I) complex-based DSSCs was further increased to 2.89% by using phosphonic acid as the anchoring group [14]. It is worth mentioning that Sandroni and co-workers enhanced the PCE of Cu(I) complex-based DSSCs to 4.66% by utilizing heteroleptic Cu(I)-based complexes combined with carboxylic acid anchoring group [15]. At present, although the PCE of Cu(I)-based DSSCs is still far behind that of Ru(II) polypyridyl complexes, it has exhibited great potential in DSSC application, and there is much room to improve the PCE of Cu(I)-based DSSCs.

In recent years, our group was devoted to the molecular design and related theoretical research of Cu(I)-based complexes for DSSCs [16,17,18,19], and a series of heteroleptic Cu(I)-based complexes with functionalized chromophores, ligands, and acceptors were investigated by the density functional theory (DFT)/time-dependent DFT (TD-DFT) approach. Furthermore, some meaningful conclusions about the internal mechanism of the effects of functional groups on the optoelectronic properties of Cu(I)-based dyes have been obtained. In addition, it is well-known that a stable sensitizer should possess an anchoring group in its molecular structure, which allows the attachment of the dye to the TiO_2_ surface. The anchoring group plays an important role in high PCE of DSSCs. However, to the best of our knowledge, only a few studies were focused on the screening and tuning of the anchoring groups to elucidate their effects on photon-to-electron performance of Cu(I)-based dyes. It is essential to enrich the research field of Cu(I)-based dyes and gain more information on the structure-property relationships, as a further step in promoting copper(I)-based complexes to effectively replace ruthenium(II) complexes in DSSCs. In this study, two groups of heteroleptic Cu(I)-based dyes, B1–B5 and P1–P5, with different anchoring groups (selected from cyanoacrylic acid, carboxylic acid, nitric acid, phosphonic acid, and sulfonic acid) in bipyridine (bpy)/2,9-dimethyl-1,10-phenanthroline (dmp) ligand were designed. The intrinsic properties, such as the electronic structure, absorption spectrum, electron excitation, and intramolecular and interfacial electron transfer, of the designed Cu(I)-based dyes were theoretically investigated by DFT and TD-DFT methods. This work aimed to shed light on how the type of anchor influenced the photoelectric properties of Cu(I)-based dyes for DSSC applications. Results of this work would provide a deep insight into the intrinsic mechanisms for the design and screening of high-performance Cu(I)-based dyes in DSSCs.

## 2. Computational Method

In this work, unless otherwise stated, all the DFT/TD-DFT calculations were performed with the Gaussian 09 program package [20]. The ground-state geometries of all the studied Cu(I)-based dyes were optimized by the B3LYP (Becke 3 parameter exchange functional with correlation functional by Lee, Yang and Parr) [21,22] exchange–correlation functional in conjunction with a mixed DZVP (double-zeta valence polarized)/6-31G(d) [23,24] basis set in DCM (dichloromethane) solvent. In this mixed basis set, the 6-31G(d) basis set was chosen for non-metal atoms, and the DZVP set supplemented with three sets of uncontracted pure angular momentum *f* functions was chosen for the Cu atom. To confirm the stationary of all the optimized geometries, vibrational analyses were also performed at the same level. The results showed that all the optimized structures corresponded to a minimum of potential energy surface with no imaginary frequency. Based on the optimized ground-state structures, electronic excitation and absorption spectra were calculated using the TD-DFT method with the 6-31G(d) basis set for C, H, O, N, and S atoms and DZVP for Cu atoms, in DCM solvent. The obtained TD-DFT results were further submitted to Multiwfn [25] to obtain the absorption spectra. In addition, the electron density differences were also calculated with Multiwfn by comparing the charge density in the ground state and excited state of the dyes. Furthermore, electron transfer parameters, including the distance of electron transfer (d^ET^), transferred charge (q^ET^), *H*, and *t* (*H* is the half of the sum of centroid axis along the electron transfer direction and *t* is the difference between d^ET^ and *H*) index were calculated with Multiwfn [25].

The solvent effect was evaluated by using the non-equilibrium implementation of the conductor-like polarizable continuum model (C-PCM) [26]. In particular, the optimized structure and solvation energy were computed by a cavity model [27] coupled to C-PCM. This approach provided results very close to those obtained by the original dielectric model for high dielectric constant solvents and thus was more efficient in geometry optimization and less prone to make numerical errors arising from the small part of the solute electron cloud lying outside the cavity [28].

For the calculation results to be close to the experimental results, calculation method calibrations were performed to find a suitable level for the excited state description of the studied Cu(I)-based dyes. Different levels of TD-DFT methods were adopted to calculate the absorption spectra of [Cu(bpy(Mes)_2_)(phen)]BF4 and [Cu(bpy(Mes)_2_)(dmp)]BF4 in DCM solution. The calculation results, together with the experimental data, are listed in Table S1 (Supplementary Materials) and plotted in Figure S1 (Supplementary Materials). As shown in Figure S1, the lowest absorption peaks were located at 476 nm (phen (1,10-phenanthroline)-based dyes) and 477 nm (dmp (2,9-dimethyl-1,10-phenanthroline)-based dyes) at the B3LYP/DZVP level, which were very close to the experimental data (476 nm and 463 nm, respectively) [29]. At the B3LYP/LanL2DZ level, both values were 450 nm for phen-based dyes and 449 nm for dmp-based dyes. At the B3LYP/6-31G(d) level, both values were 540 nm for phen-based dyes and 549 nm for dmp-based dyes, which blue-shifted severely compared with the benchmark. Comparatively, the results obtained with B3LYP/DZVP level matched better with the benchmark than those with B3LYP/LanL2DZ and B3LYP/6-31G(d) levels. Therefore, all the TD-DFT calculations were performed at the B3LYP/DZVP level unless otherwise stated.

## 3. Results and Discussions

### 3.1. Molecular Geometry

**Figure 1:**
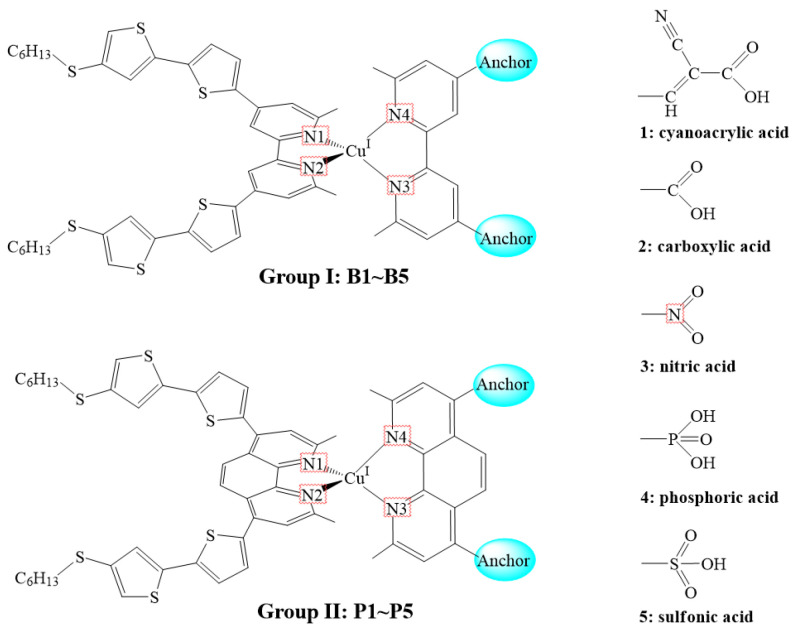
Molecular structures of B1–B5 and P1–P5.

In 2009, Chen and co-workers reported the synthesis of the Ru(II)-based dye CYC-B11 by incorporating thiophene derivatives into the ancillary ligand [30], which exhibited a high PCE of 11.5%. By referring to the CYC-B11 structure, in this study, two groups of heteroleptic Cu(I)-based dyes, B1~B5 (group I) and P1~P5 (group II), with different anchoring groups (selected from cyanoacrylic acid, carboxylic acid, nitric acid, phosphonic acid, and sulfonic acid) in bipyridine (bpy)/2,9-dimethyl-1,10-phenanthroline (dmp) ligand were designed. The structures of the designed Cu(I)-based dyes are shown in Figure 1.

The structural parameters of Cu(I)-based dyes are listed in Table 1. For all the studied dyes, the bond lengths R_Cu–N1_ and R_Cu–N2_ were in the range of 2.056–2.068 Å, while R_Cu–N3_ and R_Cu–N4_ were in the range of 2.070–2.081 Å. The bite angles of ∠N1–Cu–N2 and ∠N3–Cu–N4 fluctuated within 79.9–81.1°, while ∠N2–Cu–N3 and ∠N2–Cu–N4 fluctuated within 124.7–126.3°. The small fluctuations of bond length and bite angle indicated that the anchoring groups had a slight effect on the geometry structure of Cu(I)-based dyes. The R_Cu–N3_ and R_Cu–N4_ were obviously longer than R_Cu–N1_ and R_Cu–N2_, which indicated that the interactions between Cu(I) center and the anchoring group ligands were weaker than those between Cu(I) center and ancillary ligands. The geometry index (τ_4_) [31] is usually used to describe the four-coordinate geometry of Cu(I)-based dyes. Herein, the τ_4_ is described as

(1) $$\tau_{4}=\left\{360{}^{\circ}-\left(\theta +\varphi \right)\right\}/141{}^{\circ}$$

where *θ* and *φ* are the two largest angles in the four-coordinate geometry. The τ_4_ value is 1.00 for a perfect tetrahedron (largest angles of 109.5°), 0 for square planar (largest angles of 180°), and 0.85 for a perfect trigonal pyramid (largest angles of 120°); and intermediate geometries fall in the range of 0–1.00. As shown in Table 1, the values of τ_4_ fluctuated from 0.769 to 0.772 in group I and from 0.771 to 0.773 in group II. These results indicated that all the Cu(I)-based dyes exhibited the distorted trigonal pyramidal geometries, which were consistent with the previous studies [29]. The tiny differences of τ_4_ resulted mainly from the slight skeleton distortions of bpy and dmp ligands due to the connection of different anchoring groups.

### 3.2. Molecular Orbital and Electronic Structure

**Figure 2:**
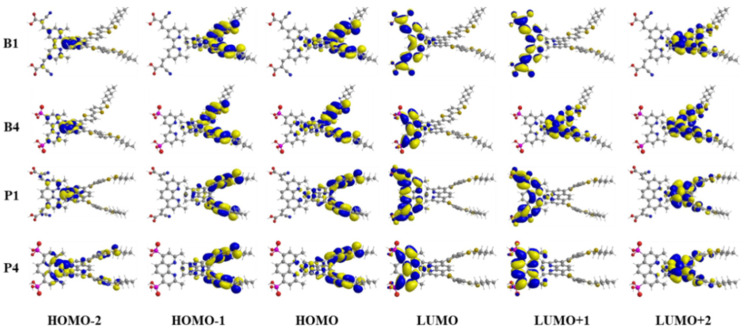
The frontier molecular orbitals from HOMO-2 to LUMO+2 of B1, B4, P1, and P4.

In DSSCs, the important electronic excitations usually occur from the highest occupied molecular orbitals (HOMOs) to the lowest unoccupied molecular orbitals (LUMOs), which significantly determine the charge-separated state of the dye. As shown in Figure 2, considering the similarity of the molecular orbital shapes, only molecular orbital plots of B1, B4, P1, and P4 were selected to discuss the frontier molecular orbital (FMO) distribution of the studied Cu(I)-based dyes. The FMO plots of the other dyes are shown in Figure S2 (Supplementary Materials). As can be seen from Figure 2 and Figure S2, all the Cu(I)-based dyes exhibited similar electron distributions on HOMOs: HOMO-2 was mainly localized at the Cu(I) center; HOMO-1 and HOMO were contributed mainly from donor subunits and only a minor percentage from the Cu(I) center. However, the electron distributions on LUMOs of Cu(I)-based dyes were diverse owing to the introduction of different anchoring groups and ligands. As shown in Figure 2 and Figure S2, for B1/P1 (with cyanoacrylic acid anchoring group), B2/P2 (with carboxylic acid anchoring group), and B3/P3 (with nitric anchoring group), the LUMO was located at the bpy/dmp moiety and anchoring group. For B4/P4 (with phosphoric anchoring group) and B5/P5 (with sulfonic anchoring group), the LUMO had fewer distribution in the anchoring group and was mainly located at the bpy/dmp moiety, which was unbeneficial for electron injection from the dye to the conduction band of the semiconductor. In addition, the LUMO+1 of dyes B2, B4, and B5 in group I, whose skeleton were based on bpy ligand, was delocalized over the Cu(I) center and donor subunits, manifesting that no efficient charge-separated state formed in these dyes and thus resulting in severe electron recombination. It is well-known that anchors play the role of adsorbing dyes onto the TiO_2_ semiconductor, so large contributions from the anchoring groups to LUMOs are valid for electron injection from dye to the conduct band of the semiconductor and can enhance the intramolecular electron transfer (IET) rate. Therefore, from the above analysis, it was not difficult to conclude that dyes with cyanoacrylic acid and nitric acid as anchoring groups exhibited more suitable FMO distribution than those with carboxylic acid, phosphoric acid, and sulfonic acid anchoring groups. In addition, compared with group I dyes (with bpy ligand), dyes with dmp ligand may exhibit a more efficient charge-separated state.

**Figure 3:**
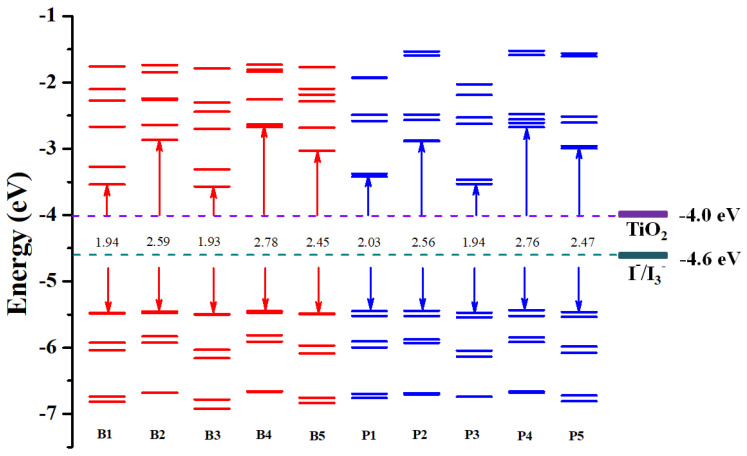
The molecular orbital energy levels from HOMO-5 to LUMO+5 and HOMO-LUMO energy gaps of all the studied Cu(I)-based dyes.

Furthermore, the energy levels of the frontier molecular orbitals from HOMO-5 to LUMO+5 and the HOMO-LUMO energy gaps of all the investigated Cu(I)-based dyes are depicted in Figure 3. As shown in Figure 3, the introduction of different anchoring groups in the molecule skeleton had a slight effect on the HOMO energy, which can be rationally understood that there were the same electron donors in the two groups of dyes. However, the LOMO levels are very sensitive to the anchoring groups. Therefore, the HOMO-LUMO energy gaps of the designed dyes can be modulated by introduction of different anchoring groups. The HOMO-LUMO energy gaps of group I fluctuated in the range of 1.93–2.78 eV, while the gaps of group II fluctuated in the range of 1.94–2.76 eV. In group I, dyes B1 (with cyanoacrylic acid anchoring group) and B3 (with nitric acid anchoring group) exhibited small HOMO-LUMO energy gaps of 1.94 eV and 1.93 eV, respectively, while B4 (with phosphoric acid anchoring group) showed the maximal gap of 2.78 eV. A similar trend was observed in group II with small HOMO-LUMO energy gaps of 2.03 eV (P1, with cyanoacrylic acid anchoring group) and 1.94 eV (P3, with nitric acid anchoring group). The results indicated that introduction of cyanoacrylic acid and nitric acid as anchoring groups in Cu(I)-based dyes could efficiently decrease HOMO-LUMO energy gaps, which would be favorable for improving the light-harvesting ability of the dyes. These will be further verified in the following discussions about absorption spectrum.

### 3.3. Absorption Spectrum and Electronic Excitation

**Figure 4:**
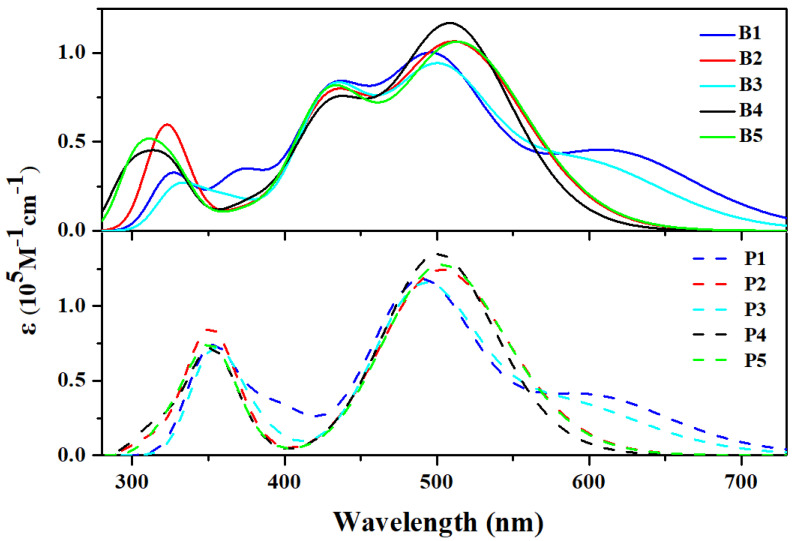
The calculated absorption spectra of all the studied Cu(I)-based dyes.

In order to investigate the light-harvesting abilities of the studied Cu(I)-based dyes, the absorption spectra of designed dyes were calculated and are displayed in Figure 4; and the related lowest excitation state parameters are listed in Table S2 (Supplementary Materials). As shown in Figure 4, all the studied Cu(I)-based dyes exhibited a similar optical absorption behavior with a short wavelength band of 260–400 nm and a long wavelength band of 400–700 nm. For the absorption peak ranges within 260–400 nm, except for P2 which showed the largest molar absorption coefficient (ε(λ)), dyes in group II showed generally identical positions and intensities; while dyes in group I, which obviously had red-shift and smaller ε(λ) compared with their counterparts in group II, showed major differences in both positions and intensities: B1–3 (centered at around ~323 nm) had red-shift compared to B4 and B5 (centered at around ~312 nm). Furthermore, for the absorption peak ranges within 400–600 nm, dyes in group II, which were centered at ~490, ~503, ~493, ~500, and ~503 nm for P1–5, respectively, showed slight blue-shift, but larger ε(λ) compared with their counterparts in group I, which were centered at ~495, ~510, ~500, ~508, and ~513 nm for B1–5, respectively. In addition, the absorption intensities of dyes in both group I and group II followed the identical sequence of B3 (P3) < B1 (P1) < B2 (P2) < B5 (P5) < B4 (P4). It is noteworthy that although B1 (P1) and B3 (P3) exhibited relatively small absorption intensities in the two main peaks, they showed better spectral response in the long wavelength region due to an absorption tail that was observed in the red region, which was centered at ~610 and ~606 nm for B1 and B3, respectively (~609 and ~600 nm for P1 and P3, respectively). This indicated that introducing cyanoacrylic acid and nitric acid as anchoring groups can broaden absorption range and thus improve the light-harvesting properties of Cu(I)-based dyes. In particular, relative to group II, all of the dyes in group I had an additional peak around ~437 nm, extending the spectral range. It was clear that dyes with bpy ligand had broader spectral coverage, while dyes with dmp ligand had stronger intensities in two major absorption peaks. Finally, it should be pointed out that the maximum absorption wavelengths of all the studied Cu(I)-based complexes had different degrees of red-shift compared with that of the calibration compounds, [Cu(bpy(Mes)_2_)(phen)]BF4 and [Cu(bpy(Mes)_2_)(dmp)]BF4, which was mainly due to the extended aromatic system in the newly designed Cu(I)-based dyes.

In order to gain more information about the light excitations in the studied Cu(I)-based dyes, the vertical excitation energies, oscillator strengths, and relative orbital contributions of the optical transitions between 400 and 650 nm for the studied Cu(I)-based dyes are listed in Table S2. Next, we began to assess the IET routes upon photo-excitations based on the excitation information from Table S2 (Supplementary Materials) and the FMO distribution from Figure 2. Considering the similarity in absorption peak and intensity, only B1, B2, and B4 (P1, P2, and P4) with the excitation spectral range over 450 nm were selected to be assessed. For dye B1, the transition patterns in the first absorption band within the range of 579.6–625.7 nm contained several typical metal-to-ligand charge transfer (MLCT) transitions (HOMO-2→LUMO/LUMO+1) and a ligand-to-ligand charge transfer (LLCT) transition (HOMO-1→LUMO+1), which was from the electron donor groups to the electron acceptor groups that could form superior charge-separated state to hinder electron recombination. Noticeably, the arriving orbitals of LUMO and LUMO+1 for these transitions were both delocalized over the whole electron acceptor groups, which was beneficial for electron injection according to the MO analysis. In addition, for the absorption band in the range of 491.8–502.0 nm in dye B1, the transitions mainly originated from HOMO/HOMO-1→LUMO+2. Unfortunately, the transitions were invalid for charge separation and electron injection since the arriving orbital of LUMO+2 had no contribution from the anchoring group. Dye P1 showed a transition behavior similar to that of B1 with two absorption bands located at ~490 and ~588 nm. The transitions of the first absorption peak mainly composed of transitions HOMO-2→LUMO (95%) with oscillator strength 0.100 at 630.6 nm and HOMO-2→LUMO+1 (86%) with oscillator strength 0.447 at 597.3 nm, which were beneficial for electron injection. However, the absorption band at ~490 nm, which was mainly composed of transitions originated from HOMO/HOMO-1→LUMO+2 in the range of 487.6–496.7 nm, were invalid for charge separation and electron injection because only LUMO/LUMO+1 had distribution on the electron acceptor subunit. Furthermore, transition behavior similar to that of B1/P1 was also found in both B3 and P3. In addition, there was one effective absorption excitation in each of the dyes B2 and P2, located at 538.2 nm with oscillator strength 0.739 and at 532.2 nm with oscillator strength 0.661, respectively. The transitions were mainly composed of HOMO-2→LUMO (55%)/HOMO→LUMO+1 (33%) for B2 and HOMO-2→LUMO (55%)/HOMO→LUMO+2 (28%) for P2, which showed typical MLCT or LLCT and were beneficial for charge separation. As for dyes B4, B5, P4, and P5, since their arriving orbitals had little contributions from the electron acceptor group, their transitions were unfavorable for electron injection from dyes to the conduction band of TiO_2_. Based on the above observations, we concluded that the anchoring groups in Cu(I)-based dyes had significant influence on electron transition and that cyanoacrylic acid and nitric acid can be used as suitable anchoring groups to generate efficient transition, thus improving IET and electron injection.

### 3.4. Excited State Lifetime

The excited state lifetime (τ) is one of the important factors to evaluate the electron transfer efficiency. A dye with a longer excited state lifetime is expected to be more susceptible to charge transfer [32]. The excited state lifetime of dyes can be evaluated by:

(2) $$\tau =1.499/{fE}^{2}$$

where *E* is the excitation energy (cm^−1^) and *f* is the oscillator strength of the excited state. According to Equation (2), the excited state lifetime (τ) was calculated and is listed in Table 2. The calculated excited state lifetime (τ) of all the dyes in group I decreased in the order of B2 (5.89 ns) > B1 (5.55 ns) > B4 (5.18 ns) > B3 (4.89 ns) > B5 (4.39 ns), and τ of all the dyes in group II decreased in the order of P4 (6.48 ns) > P2 (6.42 ns) > P1 (3.39 ns) > P3 (3.38 ns) > B5 (2.53 ns). The calculated results indicated that dyes B1/P1 (with cyanoacrylic acid anchoring group), B2/P2 (with carboxylic acid anchoring group), and B4/P4 (with phosphoric acid anchoring group) can facilitate change transfer by prolonging the excited state lifetime and may further enhance the short-circuit current density in DSSCs.

### 3.5. Intramolecular Electron Transfer

**Figure 5:**
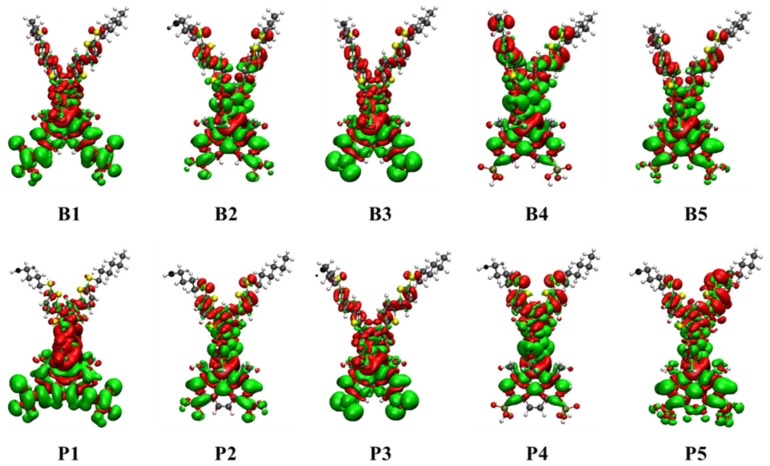
The electron density differences (Δ*ρ*) for the studied dyes. The red surface identifies the region in which the electron density decreases. The green surface identifies the region in which the electron density increases.

In this section, to further investigate the IET characteristics, the electronic density difference (EDD) plots between the ground and excited states of the studied Cu(I)-based dyes were also calculated and were shown in Figure 5. Apparently, except for B4 and P4, the region of electron density depletion (red color) for all the dyes mostly localized at the donor subunits and Cu(I) center ligand, while the region of electron density increment (green color) was largely aligned with the anchoring groups, indicating an effective charge-separated state for DSSC application. While for B4 and P4, the most majority of increased electron density was located at almost the whole complex skeleton, not on the anchor group, which would result in a serious intramolecular electron recombination and low-efficiency electron injection to the semiconductor. Among all the studied dyes, B1/P1 (with cyanoacrylic acid anchoring group) and B3/P3 (with nitric acid anchoring group) presented a superior charge-separated state, which indicated that these dyes would have great performance of electron injection and could inhibit electron recombination effectively.

Furthermore, in order to quantitatively evaluate the charge transfer characteristics of the studied Cu(I)-based dyes, charge transfer parameters, including transferred charge (q^ET^), electron transfer distance (d^ET^), *H*, and *t* of the lowest excitation states were calculated with Multiwfn [25], and the results were listed in Table 2. For more calculation details about the abovementioned parameters, please refer to these publications [33,34,35,36].

As seen in Table 2, the values of transferred charge q^ET^ fluctuated in the range of 0.604–0.687 e with the sequence of B2 < B5 < B4 < B3 < B1 in group I, while the values fluctuated in the range of 0.614–1.075 e with the sequence of P2 < P4 < P5 < P3 < P1 in group II. It was clear that dyes with cyanoacrylic acid as the anchoring group could transfer more electrons upon photo-excitation. As for the values of d^ET^, they fluctuated in the range of 1.134–3.746 Å, with the sequence of B4 < B2 < B5 < B1 < B3 in group I, while they fluctuated in the range of 1.442–3.977 Å, with the sequence of P4 < P2 < P3 < P1 < P5 in group II. The value of *t* was in the order of −4.473 Å (B4) < −3.697 Å (B2) < −3.306 Å (B5) < −1.702 Å (B1) < −1.376 Å (B3) in group I, and the same sequence was found in group II. The more positive *t* value reflected the downward trend in the orbital overlap, demonstrating a lower recombination possibility. Considering the index q^ET^, d^ET^ combined with *t*, B1/P1 (with cyanoacrylic acid anchoring group) and B3/P3 (with nitric acid anchoring group) exhibited outstanding IET characteristics. Among them, P1 performed the best due to most electrons that could be transferred with longer distance and lower orbital overlap.

### 3.6. Electron Structures of Dye/(TiO_2_)_38_ Systems

In order to investigate the interaction between dye molecules and TiO_2_ interface, an analysis of the electron injection capability of dyes and the adsorption of dyes on the anatase TiO_2_ (101) surface was performed with DFT calculations using the Dmol3 program [37,38]. Dyes B1–B5 (group I) and P1–P5 (group II) adsorbed onto the TiO_2_ surface with a bidentate bridging manner, which has been proved to be the most stable chemisorption model for the simulation of dye/TiO_2_ systems [39,40]. The dye/(TiO_2_)_38_ systems were optimized using the density functional theory (DFT) by employing the generalized gradient approximation (GGA) [41] with the Perdew–Burke–Ernzerhof (PBE) functional [42,43] and DNP (double numerical basis set with polarization) basis set.

The optimized geometries and FMOs of all the Cu(I)-based dye/(TiO_2_)_38_ systems were depicted in Table 3. The HOMOs were mainly distributed on the entire dyes, while the LUMOs were localized on the (TiO_2_)_38_ clusters except for B3, indicating excellent electron injection capacity from dyes to the TiO_2_ cluster. To further investigate the light-harvesting abilities, the values of HOMOs, LUMOs, and ΔH-L of dye-(TiO_2_)_38_ combined systems were also calculated and were listed in Table 3. The ΔH-L of group I decreased in the order of B3 > B2 > B5 > B1 > B4, and the order of group II was P5 > P3 > P4 > P1 > P2. Among them, the low ΔH-L of B4 and P2 indicated their excellent light-harvesting abilities.

It is well-known that the PCE of DSSCs can be determined by the short-circuit current density (J_sc_), open-circuit photo-voltage (V_oc_), and fill factor (FF). Next, the key parameters affecting J_sc_ and V_oc_ from the view of molecular design and evaluation will be discussed. The following expressions (3)–(6) are from references [44,45,46,47]. The V_oc_ can be described by:

(3) $$V_{OC}=\frac{E_{CB}+\Delta E_{CB}}{q}+\frac{k_{B}T}{q}ln\left(\frac{n_{c}}{N_{CB}}\right)-\frac{E_{redox}}{q}$$

where *q* is the unit charge, E_CB_ is the conduction band edge of the semiconductor substrate, ΔE_CB_ is the level shift of conduction band edge of TiO_2_ due to dye adsorption, k_B_ is the Boltzmann constant, T is the absolute temperature, n_c_ is the number of electrons in the conduction band, N_CB_ is the density of accessible states in the conduction band, and E_redox_ is the electrolyte Fermi level. ΔE_CB_ can be expressed as:

(4) $$\Delta E_{CB}=-\frac{q\mu_{\mathrm{normal}}\gamma }{\varepsilon_{0}\varepsilon }$$

where μ_normal_ denotes the dipole moment of the individual sensitizer perpendicular to the surface of the TiO_2_ semiconductor, γ is the surface concentration of dyes, and ε_0_ and ε represent the vacuum permittivity and dielectric permittivity of the dipole layer, respectively. It is obvious that a dye with larger μ_normal_ will lead to more ΔE_CB_ shift, resulting in larger V_oc_. As illustrated in Table 4, the calculated μ_normal_ values of B1–B5 (group I) were in the following order: B1 (14.99 D) > B4 (14.93 D) > B2 (12.78 D) >B5 (7.44 D) > B3 (6.95 D); and μ_normal_ values of P1–P5 (group II) were in the following order: P1 (15.95 D) > P4 (15.82 D) > P2 (13.06 D) >P3 (8.10 D) > P5 (8.09 D). The calculated results indicated that dyes B1/P1 (with cyanoacrylic acid anchoring group), B2/P2 (with carboxylic acid anchoring group), and B4/P4 (with phosphoric acid anchoring group) could display a lager μ_normal_, which would lead to more ΔE_CB_ shift, resulting in larger V_oc_ in DSSCs.

**Figure 6:**
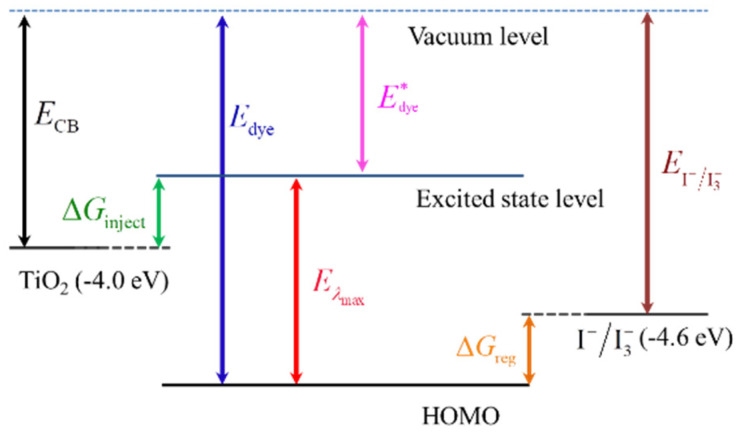
Energy level alignment of a dye-sensitized solar cell [47].

In DSSCs field, ΔG_inject_ is always used to evaluate the ability of electron injection upon photo-excitation from dyes into the conduction band of the semiconductor. As illustrated in Figure 6, ΔG_inject_ can be defined as the difference between the excited-state oxidation potential of dye (E^*^_dye_) and the conduction band energy level of semiconductor (E_CB_):

(5) $$\Delta G_{\mathrm{inject}}=E_{\mathrm{dye}}^{*}-E_{\mathrm{CB}}=\left(E_{\mathrm{dye}}-E_{\lambda_{\max}}\right)-E_{\mathrm{CB}}$$

where E_dye_ is the ground-state oxidation potential of dye, E_λmax_ is the lowest vertical transition energy corresponding to λ_max_, and E_CB_ is the reduction potential of the conduction band edge of TiO_2_, which was widely used as −4.0 eV in a previous report [48]. On the other hand, the dye regeneration efficiency in excited state is also an important factor to evaluate the performance of DSSCs, which is always estimated through the regeneration driving force ΔG_reg_. ΔG_reg_ can be calculated from the difference between the ground-state oxidation potential and redox potential of the iodide/tri-iodide redox couple. As illustrated in Figure 6, ΔG_reg_ can be expressed as:

(6) $$\Delta G_{\mathrm{reg}}=E_{I^{-}/I_{3}^{-}}-E_{\mathrm{dye}}$$

The calculated E_dye_, E^*^_dye_, ΔG_inject_, and ΔG_reg_ values for the investigated dyes are listed in Table 5. As shown in Table 5, for all the studied dyes, the absolute values of ΔG_inject_ and ΔG_reg_ were larger than 0.3 eV. It was reported that the efficient electron injection and dye regeneration process in DSSCs should require the absolute values of ΔG_inject_ and ΔG_reg_ to be at least 0.2 eV [6,49]. Therefore, we can conclude that the energy alignment of all the investigated dye/(TiO_2_)_38_ systems could guarantee effective interface charge injection and fast dye regeneration.

The electron injection time (τ_inj_) is also a vital parameter to determine the electron transfer processes in DSSCs. The τ_inj_ can be calculated as follows [50,51]:

(7) $$\tau_{\mathrm{inj}}=658/\Delta \left(\mathrm{meV}\right)$$

(8) $$\Delta =\sum P_{i}\left|\varepsilon_{i}-E_{\mathrm{LUMO}}\left(\mathrm{ads}\right)\right|$$

where Δ is energetic broadening, *P*_i_ is the adsorbate portion of every molecular orbital, ε_i_ is orbital energy, and E_LUMO_(ads) is energy of the adsorbate’s LUMO. Based on the optimized dye/(TiO_2_)_38_ geometries, the calculated τ_inj_ of electrons from the excited state of the Cu(I)-based dyes to the conduction band of TiO_2_ was in the range of 12.81–16.59 fs, with the order of B1 (13.92) < B4 (15.78) < B5 (15.88) < B2 (16.59) < B3 (16.71) in group I, and P1 (12.81) < P4 (14.99) < P5 (15.10) < P2 (15.17) < P3 (15.27) in group II. It was clear that dyes in group II (based on dmp ligand) exhibited faster electron injection than dyes in group I (based on bpy ligand). Among all the studied Cu(I)-based dyes, B1/ P1 (with cyanoacrylic acid anchoring group) exhibited the best electron injection behavior with relative shorter τ_inj_, which would have a positive effect on the short-circuit current density (J_sc_) in DSSCs.

## 4. Conclusions

In this study, two groups of heteroleptic Cu(I)-based dyes were designed and theoretically investigated by density functional theory (DFT) and time-dependent DFT (TD-DFT) methods. Different anchors were integrated into the dye skeleton to shed light on how the type of anchor influenced the electronic structure, absorption spectrum, electron excitation, and intramolecular and interfacial electron transfer of dyes. The main points are summarized as follows:

(1) All the studied Cu(I)-based dyes were inclined to form distorted trigonal pyramidal geometries. The anchoring group had little effect on the geometry structure of the dye.

(2) All the studied Cu(I)-based dyes exhibited good light-harvesting abilities with absorption band cover in the 260–750 nm range. The results indicated that the anchoring groups can efficiently tune the spectral range as well as absorption intensity and that introducing cyanoacrylic acid and nitric acid as anchoring groups can effectively decrease the HOMO-LUMO energy gap, broaden the absorption range, and thus promote the light-harvesting properties of the Cu(I)-based dyes.

(3) Dyes with cyanoacrylic acid or nitric acid as the anchoring group (B1, B3, P1, and P3) can transfer more electrons with longer distance and weaker orbital overlap and can form favorable electron-separated state. Compared with other studied dyes, dyes with carboxylic acid as the anchoring group (B2 and P2) transferred electrons faster.

(4) Among all the studied Cu(I)-based dyes, B1(P1) with cyanoacrylic acid group exhibited the best interface electronic structure parameters with a relatively short electron injection time τ_inj_ and large dipole moment μ_normal_, which would have a positive effect on the open-circuit photo-voltage (V_oc_) and short-circuit current density (J_sc_), resulting in high PCE of DSSCs.

## Figures and Tables

**Table 1:** Calculated geometrical parameters of Cu(I)-based dyes at the B3LYP/DZVP level in DCM solution.

Parameters ^1^	B1	B2	B3	B4	B5	P1	P2	P3	P4	P5
R_Cu−N1_	2.060	2.063	2.057	2.064	2.059	2.066	2.067	2.062	2.068	2.064
R_Cu−N2_	2.059	2.063	2.056	2.064	2.059	2.066	2.067	2.062	2.068	2.064
R_Cu−N3_	2.070	2.074	2.075	2.076	2.079	2.076	2.074	2.077	2.079	2.081
R_Cu−N4_	2.072	2.075	2.077	2.076	2.078	2.077	2.074	2.078	2.078	2.081
∠N1−Cu−N2	80.8	80.6	81.0	80.6	80.9	80.9	80.8	81.1	80.8	81.0
∠N2−Cu−N3	125.6	125.6	125.7	125.6	125.8	125.3	125.3	126.2	126.0	126.3
∠N3−Cu−N4	80.0	80.2	80.0	80.0	79.9	80.4	80.1	80.0	80.4	80.2
∠N2−Cu−N4	126.0	125.6	125.6	125.8	125.7	126.0	126.0	124.9	125.2	124.7
∠N1−N2−N3−N4	80.9	81.2	81.2	81.3	81.4	80.4	80.7	82.3	82.1	82.4
*τ*_4_	0.769	0.772	0.771	0.770	0.770	0.771	0.771	0.772	0.772	0.773

**^1^** Bond lengths are in angstroms and angles are in degrees.

**Table 2:** The optical properties and intramolecular electron transfer (IET) parameters of the studied dyes.

Dyes	τ/ns	ΔH-L^2^/eV	q^ET^/e	d^ET^/Å	H/Å	t/Å
B1	5.55	1.94	0.687	3.579	5.281	−1.702
B2	5.89	2.59	0.604	1.537	5.234	−3.697
B3	4.89	1.93	0.639	3.746	5.122	−1.376
B4	5.18	2.78	0.628	1.134	5.607	−4.473
B5	4.39	2.45	0.614	2.189	5.225	−3.036
P1	3.39	2.03	1.075	3.618	4.948	−1.330
P2	6.42	2.56	0.615	2.107	5.280	−3.173
P3	3.38	1.94	1.036	3.397	4.568	−1.171
P4	6.48	2.76	0.622	1.442	5.677	−4.235
P5	2.53	2.47	0.690	3.977	6.002	−2.025

**^2^** The ΔH-L gaps were calculated according to ΔH-L = LUMO − HOMO.

**Table 3:** The optimized structures and frontier molecular orbitals (with an isodensity of 0.01 au) for dye/(TiO_2_)_38_ systems.

**Dye/(TiO_2_)_38_**	**B1/(TiO_2_)_38_**	**B2/(TiO_2_)_38_**	**B3/(TiO_2_)_38_**	**B4/(TiO_2_)_38_**	**B5/(TiO_2_)_38_**
Structure	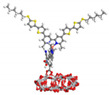	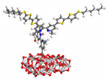	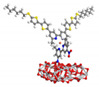	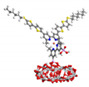	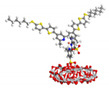
HOMO	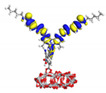	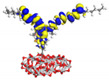	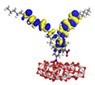	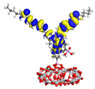	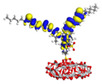
LUMO	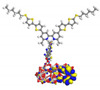	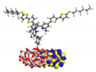	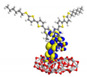	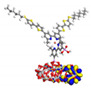	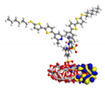
**Dye/(TiO_2_)_38_**	**P1/(TiO_2_)_38_**	**P2/(TiO_2_)_38_**	**P3/(TiO_2_)_38_**	**P4/(TiO_2_)_38_**	**P5/(TiO_2_)_38_**
Structure	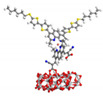	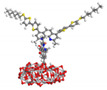	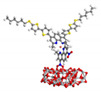	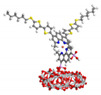	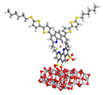
HOMO	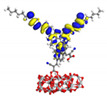	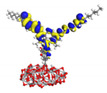	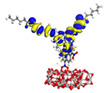	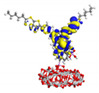	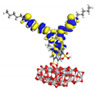
LUMO	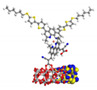	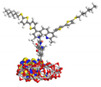	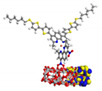	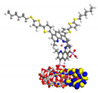	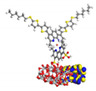

**Table 4:** Calculated energy level parameters (in eV), vertical dipole moment of the isolated dyes absorbed on (TiO_2_)_38_ clusters (in Debye), and electron injection time (in fs) for the studied dye/(TiO_2_)_38_ systems.

System	HOMO	LUMO	ΔH−L	µ_normal_	*τ*_inj_
B1	−4.87	−4.30	0.57	14.99	13.92
B2	−5.04	−4.34	0.70	12.78	16.59
B3	−5.09	−4.25	0.84	6.95	16.71
B4	−4.57	−4.24	0.33	14.93	15.78
B5	−4.82	−4.20	0.62	7.44	15.88
P1	−4.85	−4.40	0.45	15.95	12.81
P2	−4.70	−4.28	0.42	13.06	15.17
P3	−5.10	−4.26	0.84	8.10	15.27
P4	−5.04	−4.35	0.69	15.82	14.99
P5	−5.10	−4.25	0.85	8.09	15.10

**Table 5:** Parameters of interfacial electron injection and dye regeneration.

Dyes	E^dye^	λ_max_	E_λmax_	E^dye*^	ΔG_inj_	ΔG_reg_
B1	−5.42	1.98	−1.98	−3.44	0.56	0.82
B2	−5.41	2.30	−2.30	−3.11	0.89	0.81
B3	−5.47	2.05	−2.05	−3.42	0.58	0.87
B4	−5.39	2.36	−2.36	−3.03	0.97	0.79
B5	−5.44	2.29	−2.29	−3.15	0.85	0.84
P1	−5.29	1.97	−1.97	−3.32	0.68	0.69
P2	−5.38	2.33	−2.33	−3.05	0.95	0.78
P3	−5.38	1.96	−1.96	−3.42	0.58	0.78
P4	−5.33	2.36	−2.36	−2.97	1.03	0.73
P5	−5.46	2.33	−2.33	−3.13	0.87	0.86

## Supplementary Materials

The following are available online, Figure S1: Simulated absorption spectra of phen- and dmp-based dyes at the B3LYP/6-31G(d), B3LYP/DZVP, and B3LYP/LanL2DZ levels, Figure S2: The frontier molecular orbitals of dyes B2, B3, B5, P2, P3, and P5, Table S1: Calculated UV/VIS results compared with experimental and theoretical studies (nm), Table S2: The lowest excitation state parameters of all the investigated dyes.
