# Supplementary material for: Theoretical Analysis on Heteroleptic Cu(I)-Based Complexes for Dye-Sensitized Solar Cells: Effect of Anchors on Electronic Structure, Spectrum, Excitation, and Intramolecular and Interfacial Electron Transfer
Source: Molecules. 2020 Aug 12;25(16):3681. doi: 10.3390/molecules25163681 (PMC7465775; doi:10.3390/molecules25163681)
Supplement: Supplementary file 1 [file molecules-25-03681-s001.pdf]

Theoretical analysis on heteroleptic Cu(I)-based complexes for dye-sensitized solar cells: effect of anchors on electronic structure, spectrum, excitation, intramolecular and interfacial electron transfer

Zhijie Xu [1,*], Xiaoqing Lu [2,*], Yuanyuan Li [1] and Shuxian Wei [1,*]

[1] College of Science, China University of Petroleum, Qingdao, Shandong 266580, P. R. China
[2] School of Materials Science and Engineering, China University of Petroleum, Qingdao, Shandong 266580, P. R. China
* Correspondence: xuzj@upc.edu.cn (Z. X.); luxq@upc.edu.cn (X. L.); wshx@upc.edu.cn (S. W.); Telephone: +86-532-8698-3376 (Z. X.); +86-532-8698-3415 (X. L.); +86-532-8698-3410 (S. W.)

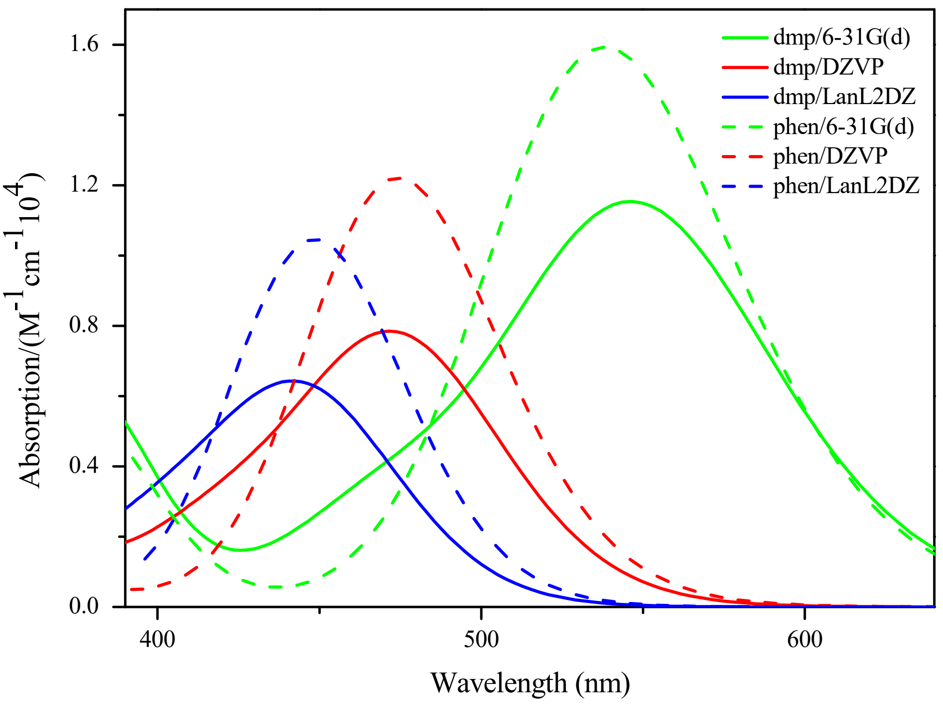

Figure S1. Simulated absorption spectra of phen- and dmp-based dyes at the B3LYP/6-31G(d), B3LYP/DZVP, and B3LYP/LanL2DZ levels.

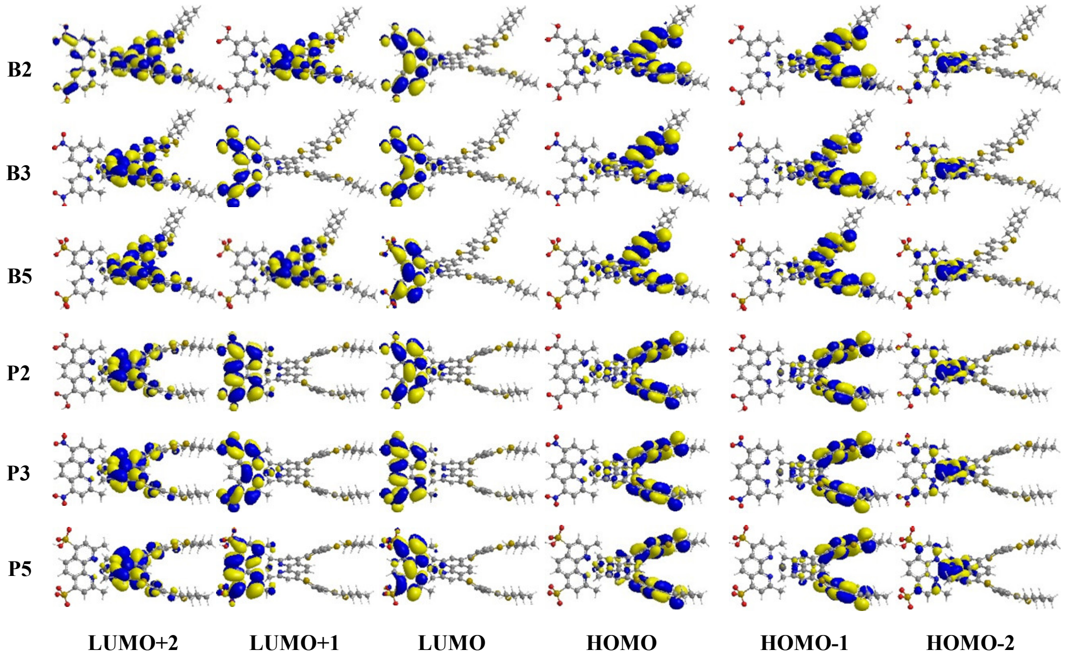

Figure S2. The frontier molecular orbitals of dyes **B2**, **B3**, **B5**, **P2**, **P3**, and **P5**.

Table S1. Calculated UV/VIS results compared with experimental and theoretical studies (nm).

Compound	Experimental	Theoretical	6-31G(d)	DZVP	LanL2DZ
$[Cu(bpy(Mes)_2)(phen)]BF_4$	476	450	540	476	450
$[Cu(bpy(Mes)_2)(dmp)]BF_4$	463	452	549	477	449

Table S2. The lowest excitation state parameters of all the investigated dyes.

Dyes	λ_{max}/nm	E/eV	f	Compositions
**B1**	625.7	1.98	0.346	H-2 → L(63%) H-1 → L+1(28%)
	617.9	2.01	0.169	H-1 → L+1(71%) H-2 → L (25%)
	579.6	2.14	0.137	H-2 → L+1(99%)
	509.3	2.43	0.179	H-5 → L(79%) H-1 → L+2(16%)
	502.0	2.47	0.680	H-1 → L+2(79%)
	491.8	2.52	0.431	H → L+2(88%)
	437.3	2.84	0.550	H → L+1(99%)
	425.4	2.91	0.523	H-1 → L+3(95%)
**B2**	538.2	2.30	0.739	H-2 → L(55%) H → L+1(33%)
	499.7	2.48	0.887	H-1 → L+1(96%)
	478.0	2.59	0.103	H → L+1(63%) H-2 → L(32%)
	439.2	2.82	0.448	H → L+2(51%) H → L+3(38%)
	423.2	2.93	0.482	H-1 → L+2(82%) H-1 → L+3(13%)
**B3**	604.0	2.05	0.450	H-2 → L(86%)
	507.9	2.44	0.791	H-1 → L+2(78%)
	491.8	2.52	0.393	H → L+2(83%)
	436.2	2.84	0.565	H → L+4(87%)
	426.7	2.91	0.529	H-1 → L+4(87%)
**B4**	525.2	2.36	0.799	H → L+1(49%) H-2 → L(38%) H-3 → L+1(10%)
	499.3	2.48	0.888	H-1 → L+1(95%)
	440.7	2.81	0.502	H → L+2(91%)
	423.0	2.93	0.475	H-1 → L+2(93%)
**B5**	541.1	2.29	0.653	H-2 → L+0(66%) H → L+1(23%)
	504.8	2.46	0.886	H-1 → L+1(95%)
	483.9	2.56	0.178	H → L+1(72%) H-2 → L+0(23%)
	436.7	2.84	0.523	H → L+2(90%)
	425.7	2.91	0.499	H-1 → L+2(94%)
**P1**	630.6	1.97	0.100	H-2 → L(95%)
	597.3	2.08	0.447	H-2 → L+1(86%)
	496.7	2.50	1.087	H → L+2(68%) H-1 → L+2(26%)
	487.6	2.54	0.328	H → L(78%)
	459.5	2.70	0.316	H-1 → L+3(97%)
**P2**	532.2	2.33	0.661	H-2 → L+0(55%) H → L+2(28%)
	496.6	2.50	1.083	H → L+3(74%) H-1 → L+2(22%)
	457.5	2.71	0.302	H-1 → L+3(94%)
**P3**	632.2	1.96	0.046	H-2 → L+0(96%)
	586.7	2.11	0.417	H-2 → L+1(85%)
	498.6	2.49	1.099	H → L+3(61%) H-1 → L+2(35%)
	491.9	2.52	0.255	H → L+2(79%)
	462.4	2.68	0.306	H-1 → L+3(97%)
**P4**	518.3	2.39	0.623	H-0 → L+2(43%) H-2 → L(32%) H-2 → L+2(14%)
	508.6	2.44	0.122	H-2 → L+2(79%)

	496.3	2.50	1.065	H-0 → L+3(75%) H-1 → L+2(21%)
	456.2	2.72	0.277	H-1 → L+3(87%)
**P5**	532.9	2.33	0.359	H-2 → L+0(28%) H-3 → L+0(25%) H-0 → L+0(18%)
				H-0 → L+2(13%)
	527.9	2.35	0.165	H-1 → L+1(42%) H-2 → L+0(26%)
	525.6	2.36	0.129	H-2 → L+1(82%)
	497.5	2.49	1.119	H-0 → L+3(67%) H-1 → L+2(31%)
	460.4	2.69	0.294	H-1 → L+3(95%)

Note: Only oscillator strength f > 0.01 and orbital percentage > 10% are reported, where H = HOMO and L = LUMO.
